# Physiological basis for muscle stiffness and weakness in a knock‐in M1592V mouse model of hyperkalemic periodic paralysis

**DOI:** 10.14814/phy2.12656

**Published:** 2015-12-23

**Authors:** Shiemaa Khogali, Brooke Lucas, Tarek Ammar, Danica Dejong, Michael Barbalinardo, Lawrence J. Hayward, Jean‐Marc Renaud

**Affiliations:** ^1^ Department of Cellular and Molecular MedicineUniversity of OttawaOttawaOntarioCanada; ^2^ Department of NeurologyUniversity of Massachusetts Medical SchoolWorcesterMassachusetts

**Keywords:** Calcium, EMG, potassium, tetanic force

## Abstract

The mechanisms responsible for the onset and progressive worsening of episodic muscle stiffness and weakness in hyperkalemic periodic paralysis (HyperKPP) are not fully understood. Using a knock‐in HyperKPP mouse model harboring the M1592V Na_V_1.4 channel mutant, we interrogated changes in physiological defects during the first year, including tetrodotoxin‐sensitive Na^+^ influx, hindlimb electromyographic (EMG) activity and immobility, muscle weakness induced by elevated [K^+^]e, myofiber‐type composition, and myofiber damage. In situ EMG activity was greater in HyperKPP than wild‐type gastrocnemius, whereas spontaneous muscle contractions were observed in vitro. We suggest that both the greater EMG activity and spontaneous contractions are related to periods of hyperexcitability during which fibers generate action potentials by themselves in the absence of any stimulation and that these periods are the cause of the muscle stiffness reported by patients. HyperKPP muscles had a greater sensitivity to the K^+^‐induced force depression than wild‐type muscles. So, an increased interstitial K^+^ concentration locally near subsets of myofibers as a result of the hyperexcitability likely produced partial loss of force rather than complete paralysis. Na_V_1.4 channel protein content reached adult level by 3 weeks postnatal in both wild type and HyperKPP and apparent symptoms did not worsen after the first month of age suggesting (i) that the phenotypic behavior of M1592V HyperKPP muscles results from defective function of mutant Na_V_1.4 channels rather than other changes in protein expression after the first month and (ii) that the lag in onset during the first decade and the progression of human HyperKPP symptoms during adolescence are a function of Na_V_1.4 channel content.

## Introduction

Hyperkalemic periodic paralysis (HyperKPP) is an autosomal dominant disease with complete or nearly complete penetrance (Gamstorp et al. 1957; Pearson 1964; Bradley et al. 1990). In general, affected individuals suffer of muscle stiffness, with myotonic discharges in some but not all cases, occurring during and between paralytic attacks causing weakness (Lehmann‐Horn et al. 1983; Bradley et al. 1990; Miller et al. 2004; Jurkat‐Rott and Lehmann‐Horn 2007). A key hallmark of the disease is the precipitation of paralytic attacks following potassium ingestion (Gamstorp et al. 1957; Poskanzer and Kerr 1961; Wang and Clausen 1976). Paralysis is in some but not in all cases associated with an increased plasma [K^+^] from 4 to 6–8 mmol/L (Gamstorp et al. 1957; Poskanzer and Kerr 1961; Lehmann‐Horn et al. 1983; Rüdel and Ricker 1985; Chinnery et al. 2002; Miller et al. 2004).

HyperKPP is linked to missense mutations in the SCN4A gene that encodes for the *α*‐subunit of NaV1.4 Na^+^ channel, the Nav isoform expressed in adult skeletal muscle (Zhou and Hoffman 1994; Cannon 2006). Patients with the T704M mutation are reported to suffer between 8 and 42 paralytic attacks per month compared to 5–6 attacks for the M1592V mutation, whereas the mean duration of paralysis is about 10 times longer for the M1592V mutation than for the T704M (89 h vs. 8 h) (Miller et al. 2004). A study of an English family with 21 affected individuals (Poskanzer and Kerr 1961), later found to carry the M1592V mutation (Chinnery et al. 2002), reported patients being bedridden because of complete limb paralysis occurring once or twice a year. More typically, patients suffer milder attacks (4–5 days in duration) consisting of muscle stiffness and weakness without any sign of myotonia. During an attack, patients remain mobile, but are severely impaired in activity. For one individual, myotonic discharges were observed in all tested muscles, but the discharges were elicited upon penetration of the EMG concentric needle into the muscle (Chinnery et al. 2002). It is therefore not clear as to whether muscle stiffness really occurs as a result of myotonic discharges as defined clinically (i.e., following a contraction (Cannon 2006) or from the generation of action potentials by muscle fibers themselves at any time during the day. So, one objective was to better understand whether the underlying mechanisms contributing to muscle stiffness in HyperKPP patients involve myotonic discharge.

Factors influencing the onset and progression of HyperKPP are also inadequately understood. For the M1592V mutation, the attacks usually start between the ages of 5 and 10 years, becoming longer and more severe during adolescence (Poskanzer and Kerr 1961; Miller et al. 2004). Considering the difference in human and mouse lifespan, the second objective was to provide answers to three questions: (i) how are the symptoms progressing in the M1592V HyperKPP mouse model?; (ii) at what ages should we study the disease in the mouse model?; (iii) is the severity of HyperKPP symptoms in terms of membrane excitability defects linked to the NaV1.4 mutant expression or does it continue to worsen after the channel content has reached adult level? The last question is important for two reasons. First, in human, the NaV1.4 mRNA content at age 5 is only 30% of the adult level (Zhou and Hoffman 1994). It is therefore possible that the symptom worsening during adolescence occurs as the Na_V_1.4 channel content increases to adult level. Second, findings also indicate changes in the expression of myosin isoforms, metabolic enzymes, PGC‐1*α*, NaV1.4 channels, and the Na^+^ K^+^ ATPase pump in HyperKPP muscles (Hayward et al. 2008; Clausen et al. 2011; Lucas et al. 2014) and it is therefore possible that some of these changes in protein expression are also responsible for the symptom worsening.

To gain a more complete understanding of mechanisms relevant to HyperKPP and to answer the above questions, we assessed how Na^+^ influx, electromyographic (EMG) activity, hindlimb immobility, force generation at different [K^+^]_e_, muscle fiber type composition, and myofiber damage change as a function of NaV1.4 protein content during the first 12 months of age in the M1592V HyperKPP mouse model. The M1592V HyperKPP mouse model was used because it has so far recapitulated many of the abnormalities observed in human patients including increased Na^+^ influx, greater membrane depolarization, and increased sensitivity to the K^+^‐induced force depression (Hayward et al. 2008; Clausen et al. 2011; Lucas et al. 2014; Ammar et al. 2015).

## Materials and Methods

### Animals

HyperKPP mice were generated by knocking in the equivalent human missense mutation M1592V in the genome of FVB1N mice; that is a mutation of methionine to valine at position 1585 (Hayward et al. 2008). The homozygous mutants do not survive past postnatal day five (Hayward et al. 2008). HyperKPP mice were generated by crossbreeding heterozygous HyperKPP mice with FVB mice. All wild type and HyperKPP mice used in this study were those obtained by continuously crossbreeding FVB and HyperKPP mice. All mice were fed ad libitum and housed according to the guidelines of the Canadian Council for Animal Care. The Animal Care Committee of the University of Ottawa approved all experimental procedures used in this study. Prior to any EMG electrodes transplantation or muscle excision, mice were anaesthetized with a single intraperitoneal injection of 2.2 mg ketamine/0.4 mg xylazine/0.22 mg acepromazine per 10 g of animal body weight. All mice were sacrificed by cervical dislocation while under anesthesia.

### Genotyping

A 2‐mm tail piece was incubated overnight with 500 *μ*L tail digestion buffer (0.2 mmol/L Na_2_EDTA and 25 mmol/L NaOH, pH 12.3) and 50 *μ*L Proteinase K (1 mg/mL) at 56°C. DNA extraction involved the addition of 650 *μ*L of 1:1 Phenol:chloroform:isoamyl alcohol (24:1; CIA) and centrifuged at 12,000 ***g*** for 10 min. Twice, 650 *μ*L of CIA was added to the pellet and centrifuged before suspending the resulting pellet in 750 *μ*L of isopropyl alcohol. After 10 min, the solution was centrifuged 15 min at 15,000 ***g***. The alcohol was removed and the pellet suspended in 750 *μ*L 70% ethanol and centrifuged. After removing the alcohol, the pellet was let to dry 30 min prior to addition of 200 *μ*L TE buffer (10 mmol/L Tris, 1 mmol/L EDTA, pH 8.0) and incubated at 65°C for 2 h. PCR was then completed using the previously extracted DNA and the following primers:

NC1F (forward): 5′ TGT CTA ACT TCG CCT ACG TCA A 3′
NC2R (reverse): 5′ GAG TCA CCC AGT ACC TCT TTG G 3′

PCR products were digested 6 h using the restriction digest enzyme *Nsp*I. The mutation that is knocked in to the HyperKPP mice causes the removal of the *Nsp*I splice site; so on an electrophoresis gel, two bands were visualized for wild‐type mice, which carry the splice site on both alleles and three bands was seen for HyperKPP mice because being heterozygotes they have one normal allele and one mutated allele.

### NaV1.4 Western Blots

Muscles were homogenized in buffer containing (in mmol/L): 50 Tris, 150 NaCl, 1% Triton X‐100, 0.5% sodium deoxycholate 0.1% SDS, protease inhibitor cocktail, and pH 8.0. Homogenates were kept 10 min on ice then centrifuged 15 min at 16,000 ***g*** and 4°C. Protein concentration was determined in supernatants using the BC assay method (Thermo Scientific, Montreal, QC, Canada). Forty *μ*g of proteins were resolved on a 6% of acrylamide gel at 100 V and then transferred onto nitrocellulose membranes (Miniprotean III apparatus; Bio‐Rad Laboratories Inc., Mississauga, ON, Canada). Total protein contents on membrane were verified with Ponceau S (MP Biomedicals, Illkirch, France). Membranes were blocked overnight at 4°C with 5% skim milk powder in phosphate‐buffered solution (PBS) containing 0.1% Tween, washed three times (10 min each) with PBS and incubated 2 h with rabbit anti Nav1.4 (1:200 dilution; Alomone labs Ltd, Jerusalem, Israel) in the presence of 5% skim milk, 1% bovine serum albumin (BSA) in PBS. Membranes were washed three times (10 min each) with PBS and incubated 1 h with horseradish peroxidase conjugated goat anti‐rabbit antibody (Jackson ImmunoResearch Laboratories, Inc., West Grove, PA) diluted 1:10,000 in 5% skim milk in PBS. Bands were visualized by chemiluminescence using the ECL kit (PerkinElmer, Inc., Waltham, MA) on Cl‐X Posure film (Thermo Scientific). Films (Cl‐XPosure film; Thermo Scientific) were scanned (MP 600 Cannon PIXMA, Mississauga, ON, Canada) and quantified using Image J (U.S. National Institutes of Health, Bethesda, MD). When comparing the NaV1.4 channel protein content between the same muscles, *β*‐actin band was visualized using horseradish peroxidase‐conjugate mouse monoclonal anti‐*β*‐actin antibody (dilution 1:2000; Santa Cruz Biotechnology, Inc., Dallas, TX).

### Treadmill running

Mice ran on treadmill with a 15° uphill angle. The initial speed was set at 10 m/min and incremented every 5 min by 5 m/min until it reached a maximum speed of 25 m/min. No electrical shock was used. Instead, mice were elicited to run by a gentle touch at the base of the tail or with a sponge placed midway in the lane to trigger an escape reaction. Mice were removed from the treadmill when they could no longer maintain the required speed. The total distance ran was then calculated from the time spent at each speed and running speed.

### EMG measurements

Three 2‐mm‐long platinum electrodes were implanted: two in the gastrocnemius, and one at the mid back (at a position that did not pick up the cardiac electrical activity). The electrodes in the gastrocnemius were threaded under the skin via an incision at the back of the head with the help of a trocar. The third electrode was also threaded under the skin into the mid back. The electrodes were secured to the skin with two sutures and tissue glue. Skin incisions were closed with tissue glue and sutures. Mice were allowed to recover for 1 h and EMG electrodes were connected to a Grass P55 AC preamplifier (Grass Technologies/Astro‐Med Inc., Warwick, RI) plugged to a data acquisition system (model KCPI3104; Keithley, Cleveland, OH). EMG signals were acquired at 10 kHz (fast EMG signals were lost if acquisition was below 5 kHz). The integrated EMG (iEMG) was calculated by computer analysis as follows: the original EMG trace (Fig. 4A) was first rectified (Fig. 4B) and iEMG was calculated from the cumulative area under the trace (Fig. 4C). To verify that leg movement did not give rise to any electrical artifacts, mice were anesthetized at the end of the measuring period; the EMG signal became completely silent in all gastrocnemius even when the leg was vigorously moved (see inset of Fig. 4A).

### Physiological measurements in vitro

#### Physiological solutions

Control physiological saline solution contained (in mmol/L): 118.5 NaCl, 4.7 KCl, 2.4 CaCl_2_, 3.1 MgCl_2_, 25 NaHCO_3_, 2 NaH_2_PO_4_, and 5.5 d‐glucose. Solutions containing different K^+^ or Ca^2+^ concentrations were prepared by adding the appropriate amount of KCl or CaCl_2_, respectively. Tetrodotoxin (TTX, a Na^+^ channel blocker) and ouabain (Na^+^ K^+^ pump inhibitor; Sigma, Oakville, ON, Canada) were prepared by adding them directly in the control solution. All solutions were continuously bubbled with 95% O_2_–5% CO_2_ to maintain a pH of 7.4. Experimental temperature was 37°C.

#### Na^+^ influx

Muscles were mounted vertically into chambers consisting of methyl acrylate cuvettes. Muscles were equilibrated 30 min in 2 mL physiological solution before being incubated 10 min with 2 *μ*Ci/mL ^22^Na^+^. Extracellular ^22^Na^+^ was washout out as described by Clausen et al. (2011). Briefly, muscles were bathed once for 5 min and three times for 15 min in Na^+^‐free solutions containing (in mmol/L) 10 mmol/L HEPES, 280 mmol/L sucrose, 500 nmol/L TTX, pH7.4. TTX was added to prevent loss of intracellular ^22^Na^+^ during washout, especially in HyperKPP muscles. The washing period consisted of three 15‐min‐long wash which was long enough to remove all extracellular ^14^C‐sucrose in another experiment (data not shown). ^22^Na^+^ was counted using a LKB Wallac gamma counter (1282 Compugamma, Mount Waverley, Vic., Australia).

#### Force measurement

Muscles were positioned horizontally in a Plexiglas chamber containing 5 mL of physiological solution. One end of the EDL was attached to a force transducer (Model # 400A; Aurora Scientific, Aurora, ON, Canada), whereas the other end was fixed to a stationary hook. Flow of physiological solution below and above muscles was maintained at a total of 15 mL/min. Muscle length was adjusted to give maximal tetanic force. Tetanic contractions were elicited with electrical stimulations applied across two platinum wires (4 mm apart) located on opposite sides of the fibers. Electrodes were connected to a Grass S88 stimulator and a Grass SIU5 isolation unit (Grass Technologies/Astro‐Med Inc.). Tetanic contractions were elicited every 100 ses with 200 ms trains of 0.3 ms, 12 V (supramaximal voltage) pulses; stimulation frequencies for soleus and EDL were, respectively, 140 and 200 Hz.

Force transducers were connected to the same KCP13104 data acquisition system described above and data were recorded at 5 kHz. Parameters of the tetanic contraction were then later analyzed with a computer. Tetanic force was defined as the maximal force generated when muscles were elicited to contract by electrical stimulation and was calculated as the difference in force between the peak force and the averaged force 5 msec prior to stimulation. Unstimulated force was defined as the force exerted by muscles in the absence of electrical stimulation and was calculated as the difference in baseline and zero force.

### Histological measurements

#### Hematoxylin and eosin staining

This staining was used to determine the extent of any fiber damage in EDL muscles. Hematoxylin and eosin staining staining was carried as previously described (Thabet et al. 2005). Briefly, muscles were embedded in Tissue‐Tek^®^ Optimum Cutting Temperature (Sakura Finetek, Torrance, CA) compound and frozen in isopentane precooled in liquid nitrogen. Ten‐*μ*m‐thick cross sections were cut from the mid‐belly of each muscle using a cryostat (Leica Microtome, HM 500M, Concord, ON, Canada) and mounted on Superfrost Plus slides. Cross sections were incubated in hematoxylin solution (Shandon), washed dipped in eosin solution (Shandon, Thermo Scientific), mounted in Permount before the coverslip was placed over the sample.

#### Myosin isoform expression

Immunohistochemical analysis was used to determine the expression of the different myosin isoforms. Serial cross sections were double‐stained with rabbit anti‐laminin antibody (Sigma) and one of mouse monoclonal anti‐MHC I (A4.840), IIA (SC‐71), IIB (BF‐F3) or IIX (6H1) antibodies (Developmental Studies Hybridoma Bank, Iowa City, IA). Anti‐laminin antibody was used to better identify boundaries between individual fibers. For myosin MHC‐I, MHC‐IIA, and MHC‐IIB staining, slides were placed were placed on a 37°C slide warmer for 15 min before being rinsed 3 min in PBS. Nonspecific binding was blocked for 1 h at RT in a humid chamber with 0.5% BSA in PBS before being exposed to primary antibodies for 2 h at room temperature in a humid chamber. Sections were rinsed in PBS three times (5 min each time) before a 45 min incubation with secondary antibodies diluted in triton‐X 100 at 37°C in a humid chamber for 2 h. Secondary antibodies were as follows: rhodamine‐conjugated goat anti‐rabbit IgG for laminin; FITC‐conjugated goat anti‐mouse IgG for myosin IIA and IgM for myosin I and IIB. Sections were rinsed three times in PBS, mounted with anti‐fade reagent, and stored at −80°C until viewed. Control sections were also stained without the primary antibody to test for nonspecific secondary antibody binding.

For MHC‐IIX, the staining protocol was slightly modified to enhance primary antibody binding and block nonspecific binding. After sections were thawed on the slide dryer, they were fixed in 100% ethanol for 5 min then rinsed in PBS that was kept at pH of 7.4. Sections were blocked with 5% horse serum (HS) in PBS, and incubated overnight at 4°C with anti‐myosin IIX and anti‐laminin antibodies. Sections were rinsed three times in PBS (5 min each time) and incubated for 1 h with the secondary antibodies diluted in 5% HS blocking solution. Sections were rinsed in PBS, mounted with anti‐fade reagent, and kept at −80°C until viewed.

### Statistical analysis

Data are presented as means ± standard error (SE). Analyses of variance (ANOVA) was used to determine significant differences. Split plot ANOVA designs were used when mice or muscles were tested at all times or K^+^ levels. ANOVA calculations were made using the version 9.2 GLM (General Linear Model) procedures of the Statistical Analysis Software (SAS Institute Inc., Cary, NC). When a main effect or an interaction was significant, the least square difference (LSD) was used to locate the significant differences (Steel and Torrie 1980). The word “significant” only refers to a statistical difference (*P* < 0.05).

## Results

### NaV1.4 protein content

**Figure 1:**
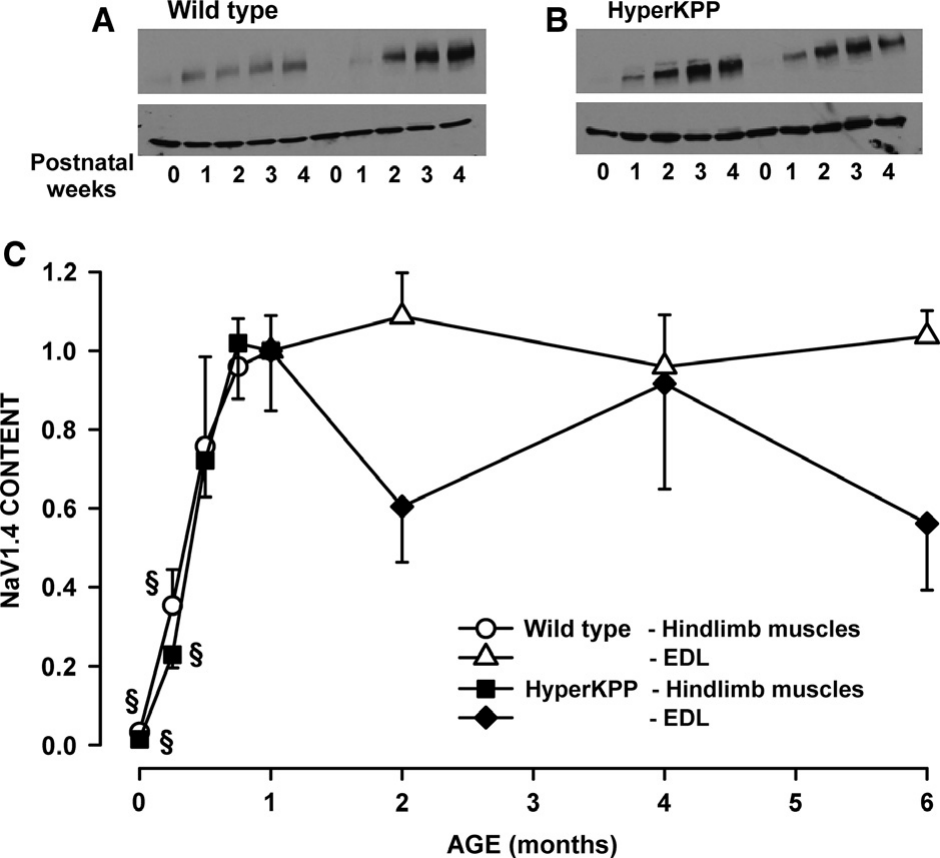
The total Nav1.4 protein content reached adult level by 3 weeks of age in both wild type and HyperKPP muscles. Examples of Western Blots from (A) wild‐type FVB and (B) HyperKPP muscles. Top: NaV1.4; bottom: *β*‐actin. (C) Changes in total Nav1.4 content. For the ages of 0–4 weeks, Nav1.4 protein content was determined using all hindlimb muscles to have sufficient proteins for Western Blot. For the ages from 1‐ to 6‐month old, NaV1.4 channel content was determined only in the symptomatic EDL. Nav1.4 content was first expressed as a ratio of *β*‐actin content before being converted as a ratio of the data at 1 month. Vertical bars represent the SE of five muscles (age 0–4 weeks), five wild type, and 8–9 HyperKPP EDL (age 1–6 months). There was no significant difference between wild type and HyperKPP (ANOVA, *P* > 0.05). ^§^Mean NaV1.4 content was significantly different from the mean content at 1 month, ANOVA, and LSD *P* < 0.05. LSD, least square difference; ANOVA, analyses of variance; HyperKPP, hyperkalemic periodic paralysis.

Skeletal muscles from heterozygous HyperKPP mice harboring one normal and one mutant *SCN4A* allele previously were shown to express mRNAs encoding both normal and mutant Na_V_1.4 channels, the latter comprising 42% of the total Na_V_1.4 mRNA transcripts (Hayward et al. 2008). However, the absence of antibodies or toxins specific for the mutant channel precludes a direct measurement of the normal versus mutant Na_V_1.4 channel contents. Alternatively, we investigated how the presence of the mutant channel alters the total Na_V_1.4 content. We previously reported that the Na_V_1.4 protein content of different adult HyperKPP muscles was 54–73% of the content in their wild‐type counterparts (Lucas et al. 2014). At the time we measured the differences in Na_V_1.4 channel content between muscles and mice, we had also assessed whether or not the increases in Na_V_1.4 protein content following birth differs between wild type and HyperKPP; data which are now reported here. The Na_V_1.4 protein content was very low at birth and reached a maximum by 3 weeks of age for both wild type and HyperKPP leg muscles (Fig. 1). Thereafter, the Na_V_1.4 protein content of wild‐type muscle remained unchanged between 1 and 6 months of age. Notably, a large variability in Na_V_1.4 content was observed between the age of 2 and 6 months as well as between samples for a given month, the latter resulting in large SE. This variability could not be explained but suggests that there might be some anomaly in the cell membrane.

### Na^+^ influx

**Figure 2:**
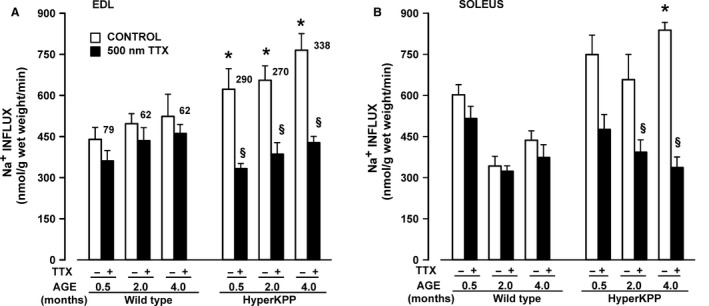
TTX‐sensitive ^22^Na^+^ influx was significantly greater in HyperKPP than in wild‐type muscles. ^22^Na^+^ influx was measured in the absence and presence of 500 nmol/L TTX using paired EDL and solei. Numbers beside each white bar represent the difference in mean ^22^Na^+^ influx with and without TTX. Vertical bars represent the SE of five muscles. ^§^Mean Na^+^ influx in the presence of TTX was significantly different from the mean value in the absence of TTX, ANOVA, and LSD *P* < 0.05. *Mean Na^+^ influx of HyperKPP muscles was significantly different from that of wild type, ANOVA, and LSD *P* < 0.05. LSD, least square difference; ANOVA, analyses of variance; TTX, tetrodotoxin; HyperKPP, hyperkalemic periodic paralysis.

A major physiological consequence of the M1592V mutation is a significant increase in TTX‐sensitive Na^+^ influx when muscles are at rest (Clausen et al. 2011; Lucas et al. 2014). The aim here was to determine how the magnitude of the Na^+^ influx changes with aging. Total Na^+^ influxes in wild‐type EDL ranged between 440 and 524 nmol/g wet weight per min in EDL from wild‐type mice that were 0.5–4 months of age (Fig. 2A). The addition of 500 nmol/L TTX, to block NaV1.4 channels, caused only a slight reduction of 62–79 nmol/g wet weight per min. In HyperKPP EDL, total Na^+^ influxes were significantly greater than those in wild‐type EDL, being 622–765 nmol/g wet weight per min. Exposure to TTX significantly reduced Na^+^ influx to levels similar to those observed in wild‐type EDL. The TTX‐sensitive Na^+^ influx in HyperKPP EDL ranged from 270 to 338 nmol/g wet weight per min; the highest mean value was at 4 months old, being 16% above the mean value at 0.5 and 2 months. The situation was very similar with soleus (Fig. 2B). That is, the TTX‐sensitive Na^+^ influx was much greater in HyperKPP than wild‐type soleus. There was no difference in total Na^+^ influx between the ages of 0.5–4 months, but the TTX‐Na^+^ influx at 4 months was 84% of the mean value at 0.5 and 2 months.

### Hindlimb immobility and exercise capacity

The University of Ottawa Animal Care facility staff observed during cage changes that HyperKPP mice occasionally showed hindlimb immobility manifested by dragging forward using their forelimbs only or suffered complete limb immobility upon suddenly lying on their side. In both situations, touching a mouse with a pen failed to elicit an immediate and rapid escape reaction. A major trigger for paralytic attacks in patients is rest after exercise, which can occur 70% of the time (Miller et al. 2004). To estimate the frequency of hindlimb immobility that could be induced in HyperKPP mice, we elicited them to run on a treadmill over 5 consecutive days. Mice were constantly observed during a 3‐h period each day while in their cage prior to running, during treadmill running, and back in their cage during recovery. Hindlimb immobility in HyperKPP mice occurred randomly in nonexercising and in exercising mice before, during, and after the treadmill exercise. They were brief, lasting less than 60 sec. The highest incidence of hindlimb immobility occurred in 1‐month‐old HyperKPP mice, affecting six of the seven tested mice (Table 1). For the six mice, the average number of days with at least one hindlimb immobility occurrence was 2.7 days of 5 days. At all other ages, hindlimb immobility was either a rare occurrence (age 3 weeks, 9 and 12 months old) or not observed (age 3–6 months old).

**Table 1:** The highest occurrence of hindlimb immobility in M1592V mice was observed at 1 month of age

Age (month)	Number of mice		Mean number of days for which at least one hindlimb immobility occurred (of 5 days)
	Having at least one hindlimb immobility over 5 days	Under observation	
0.75	1	6	1.0
1	6	7	2.7 ± 0.4
2	2	8	2.5 ± 0.5
3	0	10^a^	–
4	0	9^a^	–
6	0	6	–
9	2	4	1.0 ± 0.0
12	2	5	1.5 ± 0.5

Mice were observed over a 3 h period over 5 consecutive days before, during and after treadmill running. Hindlimb immobility rarely occurred and lasted less than 60 sec. The table indicates (i) the number of mice for which at least one hindlimb immobility was observed over the entire observation period and (ii) the mean number of days for which a mouse had a hindlimb immobility (no SE when only one mouse displayed hindlimb immobility). There was no significant difference between means number of days (ANOVA *P* > 0.05). ANOVA, analyses of variance.

a The proportion of mice with hindlimb immobility was significantly less than the proportion at 1 month of age, Fischer exact test, *P* < 0.05.

**Figure 3:**
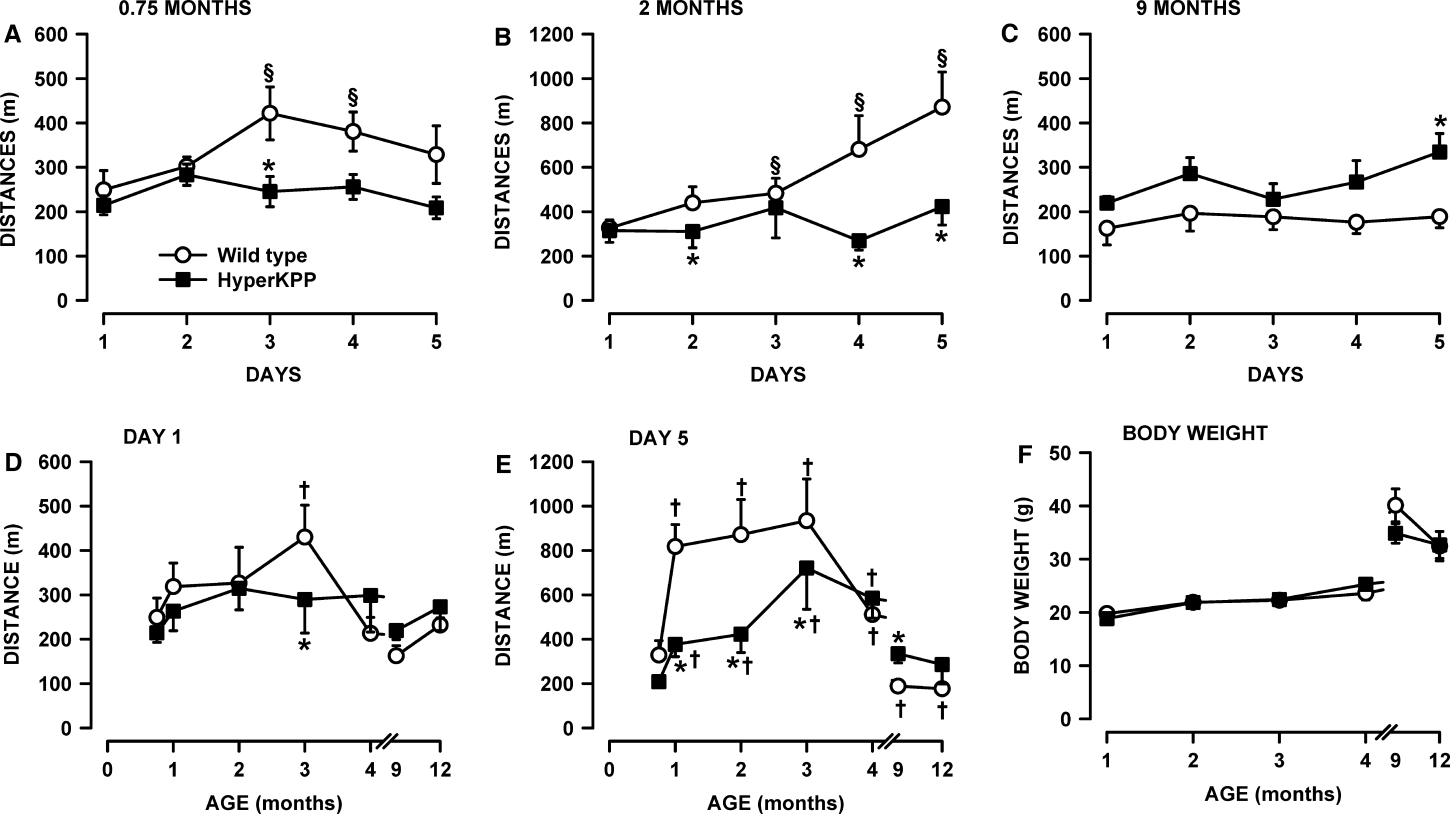
Young HyperKPP mice (1–3‐month old) have a lower running capacity than wild‐type mice while the reverse was observed for older mice (4–12 month old). Mice were elicited to run on treadmill with an uphill angle of 15° for 5 consecutive days. Each day, the initial speed was set at 10 m/min for the first for 5 min and then increased by 5 m/min every 5 min until the speed reached 25 m/min. Mice were removed from the treadmill when they could no longer maintained the required speed. Distance run over 5 days by (A) 3‐week, (B) 2‐month, and (C) 9‐month‐old mice. Effects of aging on the distance run on (D) the 1st and (E) 5th day. (F) Average body weights. Vertical bars represent the SE of 5–10 mice. ^§^Mean ran distance significantly different from the mean distance on day 1 (A–C). *Mean ran distance by HyperKPP mice significantly different from that of wild‐type mice. ^†^Mean ran distance significantly different from that at 0.75‐month‐old (D–E); ANOVA and LSD *P* < 0.05. LSD, least square difference; ANOVA, analyses of variance; HyperKPP, hyperkalemic periodic paralysis.

Three‐week‐old wild‐type mice ran 249 m on the first day of treadmill exercise before failing to maintain the required speed. Their capacity to run increased significantly to 422 m by the 3rd day and decreased to 328 m by the 5th day (Fig. 3A). No such increase in the distance run by 3‐week‐old HyperKPP mice was observed after 3 days. At 2 months of age, wild‐type mice significantly increased their running distance from 327 to 871 m by the 5th day, whereas HyperKPP mice were unable to increase their running distance significantly beyond their initial performance (Fig. 3B). Interestingly, 9‐month‐old HyperKPP mice ran longer distances (219–335 m) than wild‐type mice (163–197 m, Fig. 3C). For the first day of running, only 3‐month‐old wild‐type mice significantly ran longer distance than HyperKPP mice (Fig. 3D). On the fifth day of running, 1–3‐month‐old wild‐type mice significantly ran longer distances than HyperKPP mice (Fig. 3E) because the increase in distance ran over the 5 days was greater in wild type than HyperKPP mice. Notably, by the 4th month the reverse situation was observed in which HyperKPP mice ran longer distances than wild‐type mice. There was no difference between wild type and HyperKPP mice in mean body weights, therefore the differences in running distances between wild type and HyperKPP mice cannot be attributed to body weights (Fig. 3F).

### Electromyography

As described in the Introduction, HyperKPP is characterized by muscle stiffness with or without myotonic discharges occurring during and between paralytic attacks. Clinically, myotonia is observed when a brief contraction elicits a sustained burst of action potentials, originating from the muscle fiber itself, persisting for several seconds after motor neuron activity has ceased; whereas periodic paralysis have episodic attacks of weakness during which affected muscle fibers are electrically unexcitable (Cannon 2006). A test to detect myotonic discharges as defined above is impracticable in mouse. Hayward et al. (2008) reported that under anesthesia a myotonic discharge could without exception be elicited upon EMG needle movement, an effect not observed in wild‐type muscles; but such test gives no measure of the defect in membrane excitability during normal daily activity. As mentioned in the Introduction, patients with the M1592V mutation mainly suffer of muscle stiffness rather than myotonia per se. So here, the aim was to obtain from awaken HyperKPP mice evidence for periods of membrane hyperexcitability expected to be responsible for muscle stiffness and periods of membrane hypoexcitability expected to cause weakness or paralysis.

**Figure 4:**
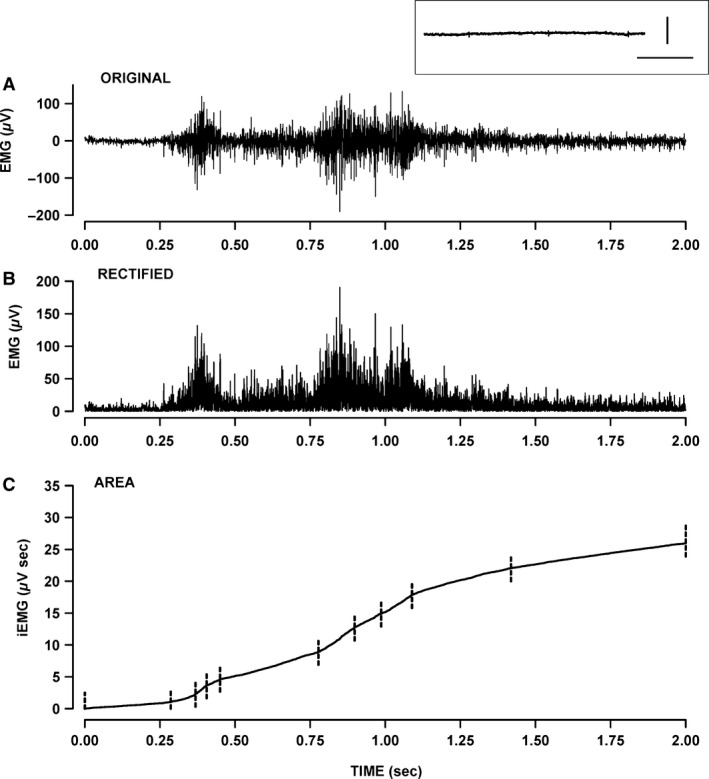
Measurement of integrated electromyographic (iEMG) from gastrocnemius. EMG was measured while the mouse moved freely in its cage. (A) Example of an original EMG trace over a 2‐sec recording. (B) Rectified EMG trace. (C) Calculated iEMG. The rate at which iEMG increased over time (diEMG/dt) varied constantly. The iEMG trace was then divided in segments as shown with the vertical dashed lines. For each segment, we calculated the slope (i.e., diEMG/dt) by linear regression analysis and its duration. Inset above A: example of an EMG trace after the mouse had been anesthetized; a similar trace was obtained if the leg was moved. Horizontal bar: 200 msec; vertical bar: 1 *μ*V.

**Figure 5:**
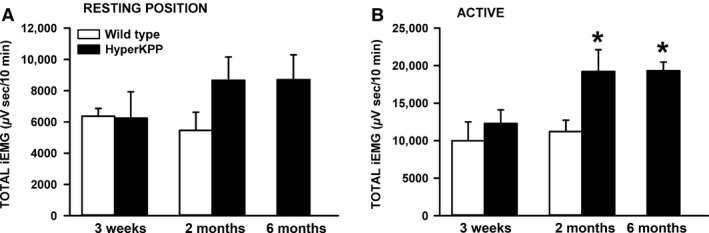
The total integrated electromyographic (iEMG) was significantly greater in HyperKPP than wild‐type gastrocnemius muscles of 2 and 6 months active mice. The total iEMG was calculated and normalized for a period of 10 min. “Resting position”: when mice were in a prone position, resting on all four limbs with no apparent movements; “Active”: when mice were standing on all four limbs and moving around. Vertical bars represent the SE of five 3‐week‐old mice, seven 2‐month‐old wild‐type mice, nine 2‐month‐old, and six 6‐month‐old HyperKPP mice. *Mean total iEMG for 2‐ and 6‐month‐old HyperKPP mice was significantly different from that of 2 months wild‐type mice, ANOVA and LSD *P* < 0.05. LSD, least square difference; ANOVA, analyses of variance; HyperKPP, hyperkalemic periodic paralysis.

To do this, we recorded the EMG of gastrocnemius muscles while mice were free to move and then calculated the iEMG and the rates at which iEMG increases over time (diEMG/dt) as shown in Figure 4. One important issue to consider is how active mice are during the measurements. In the absence of any motor control measurement, we divided the mouse activity into two categories: mice were either in a prone position, resting on all four limbs with no apparent movements (i.e., in a “resting position”) or were standing on all four limbs and moving around (i.e., being “active”). Preliminary analyses and as shown in Figure 5, the total iEMG was 2–3 times greater in the active state than in the resting position. Considering that the time mice spent in the resting position versus being active varied between mice (data not shown), the EMG data were analyzed separately for each of the two states.

In the resting position, there was no difference in iEMG between wild type and HyperKPP gastrocnemius from 3‐week‐old mice, whereas for 2‐ and 6‐month‐old mice the iEMG was 3,226 *μ*V·sec greater for HyperKPP than for 2‐month‐old wild‐type mice (Fig. 5A). In the active state, the difference between wild type and HyperKPP was again nonexistent for 3‐week‐old gastrocnemius, whereas 2‐ and 6‐month‐old HyperKPP gastrocnemius had an iEMG that was significantly greater than in wild type, the differences being 8,069 *μ*V·sec (Fig. 5B). These results suggest greater electrical activity in HyperKPP than in wild‐type gastrocnemius, especially when mice were active, but it does not reveal whether there are periods of hyper‐ and hypoexcitability that are longer in HyperKPP gastrocnemius.

A first approach to answer this question was to calculate the rate at which iEMG increases over time (i.e., diEMG/dt) as shown in Figure 4C. That is, the iEMG trace was divided in segments. For each segment, we calculated the slope (diEMG/dt) by linear regression analysis and its duration. The diEMG/dt values were then grouped in bins of 200 *μ*V·sec/min and all durations within a bin were added and expressed as a percent of the total time from all bins. From this analysis, if HyperKPP gastrocnemius has periods of hyperexcitability then the duration for the topmost diEMG/dt range should be longer in HyperKPP than in wild‐type gastrocnemius. Conversely, periods of hypoexcitability should give rise to longer duration for the smallest range of diEMG/dt (i.e., 0–200 *μ*V·sec/min) in HyperKPP gastrocnemius.

**Figure 6:**
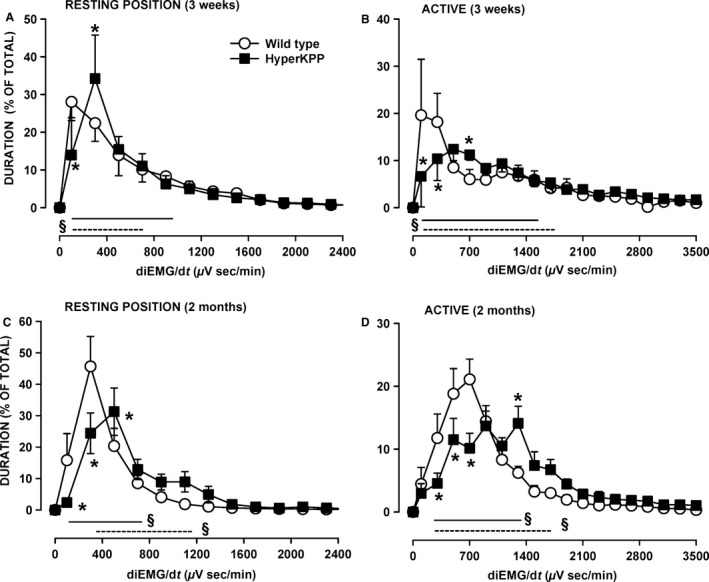
HyperKPP gastrocnemius had greater electromyographic (EMG) activity than wild type. The rates at which the integrated EMG (iEMG) increased over time (diEMG/dt) were analyzed over a 15‐min period for each of the “resting position” and “active” and separated in bin of 200 *μ*V·sec/min. The durations of all slopes within a bin were added and expressed as a percent of the total time from all bins. “Resting position”: when mice were in a prone position, resting on all four limbs with no apparent movements; “Active”: when mice were standing on all four limbs and moving around. Vertical bars represent the SE of five 3‐week‐old mice, seven 2‐month‐old wild‐type mice, and nine 2‐month‐old HyperKPP mice. ^§^Indicate with straight lines (wild type) and dashed lines (HyperKPP) when mean durations were significantly different from 0%. *Mean duration for HyperKPP mice was significantly different from that of wild type, ANOVA and LSD *P* < 0.05. LSD, least square difference; ANOVA, analyses of variance; HyperKPP, hyperkalemic periodic paralysis.

In the resting position, gastrocnemius from 3‐week‐old wild‐type mice had diEMG/dt in the 0–200 *μ*V·sec/min range 28% of the time, a value that was significantly greater than the 14% for HyperKPP (Fig. 6A). The situation was reverse for the 200–400 *μ*V·sec/min range as the values were 22% and 34%, respectively. No difference between wild type and HyperKPP was observed at diEMG/dt above 400 *μ*V·sec/min. When mice were active, diEMG/dt in the 0–400 *μ*V·sec/min range was observed 38% of the time compared to only 18% for HyperKPP (Fig. 6B). Then, for the 400–1000 *μ*V·sec/min range the values were 21% for wild type and 32% for HyperKPP, with no further differences when diEMG/dt was above 1000 *μ*V·sec/min. So, gastrocnemius of 3‐week‐old HyperKPP mice had a small tendency to maintain higher diEMG/dt more often during the day when compared with wild type.

This tendency was more pronounced at 2 months of age. In the resting position, gastrocnemius HyperKPP rarely had diEMG/dt in the 0–200 *μ*V·sec/min range (<3% of the time) compared to 16% for wild type (Fig. 6C). Even for the 200–400 *μ*V·sec/min range, the values were still smaller in HyperKPP than wild type, being, respectively, 24% and 46%. When active, the time when diEMG/dt was between 200 and 800 *μ*V·sec/min was 52% of total in wild type and only 26% for HyperKPP (Fig. 6D). Then in the 1200–2000 *μ*V·sec/min, the time values were 14% for wild type and 33% for HyperKPP. So, gastrocnemius of 2‐month‐old gastrocnemius had a significant greater tendency to maintain higher diEMG/dt more often during the day than wild type.

**Figure 7:**
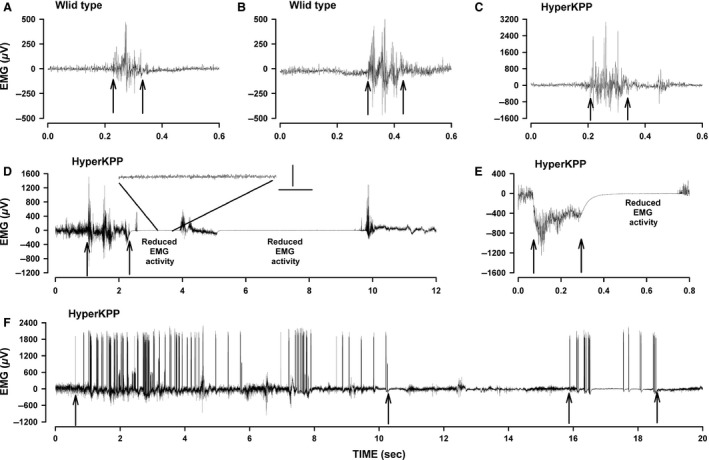
Examples of electromyographic (EMG) traces showing activity bursts and reduced electrical activity in gastrocnemius. Wild type: activity burst with a single (A) and several (B) high EMG spikes. Hyperkalemic periodic paralysis (HyperKPP): (C) activity burst with similar EMG activity before and after the burst (notice the larger EMG range for HyperKPP (C) compared to wild type (A, B)); (D) activity bursts with reduced EMG activity between bursts, with a 1 sec segment is amplified to better show the extent of the decrease in EMG, vertical bar 20 lV, horizontal bar 0.2 msec; (E) large EMG signals followed by a reduced EMG activity; (F) prolonged activity bursts. Arrows indicate the start and end of an activity burst.

In the course of the EMG analyses, differences between wild type and HyperKPP gastrocnemius were observed in regard to activity bursts. Activity bursts were defined as a single (Fig. 7A) or a series (Fig. 7B) of high EMG spikes. In HyperKPP gastrocnemius, an activity burst was observed either by itself (Fig. 7C) or was followed by a period of reduced EMG activity (Fig. 7D, E). A period of reduced EMG activity after an EMG burst was never observed in wild‐type gastrocnemius. In one occasion, a HyperKPP gastrocnemius generated two long trains of high EMG spikes; individual spike durations were less than 1 msec, whereas the spike train duration lasted about 9 sec in one case and 2.5 sec in the other (Fig. 7F). Notably, neither spike trains were followed by a period of reduced EMG activity possibly because the duration of the individual spikes were too short to cause any significant increase in [K^+^]e, the major cause for hypoexcitability of the cell membrane in HyperKPP muscles. On average, activity bursts occurred for less than 1% of the time, being similar between wild type and HyperKPP (Table 2). Mean burst duration was also similar between wild type and HyperKPP. Mean burst amplitude at 2 months of age was twofold greater in HyperKPP than in wild type. When analyzing the distribution of EMG burst amplitudes, 73% of the amplitudes ranged between 0.5 and 1.0 mV for wild type, whereas 76% of them exceeded 1.0 mV in HyperKPP and could be as high as 5 mV (data not shown). At 6 months of age, however, the mean EMG burst amplitude became significantly less than at 2 months of age for HyperKPP gastrocnemius (Table 2).

**Table 2:** Activity bursts were of similar duration but of significantly greater amplitude in gastrocnemius from 2‐month‐old HyperKPP mice than in 2‐month‐old wild type and 6‐month HyperKPP mice while reduction in EMG activity after an activity burst was predominantly observed in HyperKPP gastrocnemius

Parameters	FVB	HyperKPP	
	2 months old	2 months old	6 months old
Activity burst			
Total number analyzed	243	329	436
Time fraction (%)	0.43 ± 0.16	0.47 ± 0.08	0.53 ± 0.16
Duration (msec)	240 ± 23	260 ± 36	200 ± 24
Amplitude (mV)	1.32 ± 0.04	2.64 ± 0.46^a^	1.70 ± 0.11^b^
Reduced EMG activity			
Total number analyzed		232	10
Time fraction (%)		3.31 ± 2.04	0.11 ± 0.07
Duration (sec)		2.85 ± 0.33	0.41 ± 0.26^b^
Amplitude (*μ*V)		22.4 ± 9.9	14.2 ± 3.2

The “total number analyzed” represents the number of all activity bursts/reduced EMG activity for a period of 1 h from all mice. Time fraction represents the total duration of all activity bursts/reduced EMG activity calculated as a percent of the total time analyzed. Measurement for the duration of an activity burst is as shown with arrows in Figure 7. Amplitudes of activity bursts/reduced EMG activity were calculated as the difference from the highest and lowest EMG peak. For each parameter, an average value was calculated for each mouse and those values were used to calculate the final mean. Data are expressed as mean ± SE of five mice. EMG, electromyography; ANOVA, analyses of variance; HyperKPP, hyperkalemic periodic paralysis.

a Mean value for HyperKPP significantly different from mean value for FVB.

b Mean value for 6 month HyperKPP significantly different from mean value of 2 month HyperKPP, ANOVA *P* < 0.05.

Periods of reduced EMG activity occurred over 3.3% of the time with a mean duration of 2.9 sec and an amplitude of just 22 *μ*V in gastrocnemius of 2‐month‐old HyperKPP mice. Periods of reduced EMG activity became less frequent and were much shorter by 6 months of age, which is in agreement with the low frequency of hindlimb immobility reported in Table 1. As mentioned above, periods of reduced EMG activity usually followed an EMG activity burst. However, only 29% of activity bursts were followed by a period of reduced EMG activity (Table 3). The occurrence of reduced EMG activity appeared to depend primarily of the duration and not the amplitude of the burst as the mean burst duration that is followed by a reduced EMG activity was 2.5 times longer than those without a reduced EMG activity.

**Table 3:** Activity bursts followed by a reduced EMG activity had a similar amplitude but tended to be longer when compared to the activity bursts not followed by a reduced EMG activity

Parameter	Activity burst	
	Only	With reduced EMG activity
Number (%)	75.3 ± 6.6^a^	24.7 ± 7.0
Duration (msec)	218 ± 24	427 ± 97
Amplitude (mV)	2.64 ± 0.48	1.70 ± 0.11

The data are only for 2‐month‐old gastrocnemius as the number of reduced EMG activity periods was too small for a similar analysis from 6‐month‐old gastrocnemius. EMG, electromyography; LSD, least square difference; ANOVA, analyses of variance.

a Mean value significantly different from 50%, ANOVA and LSD *P* < 0.05.

### Contractility

**Figure 8:**
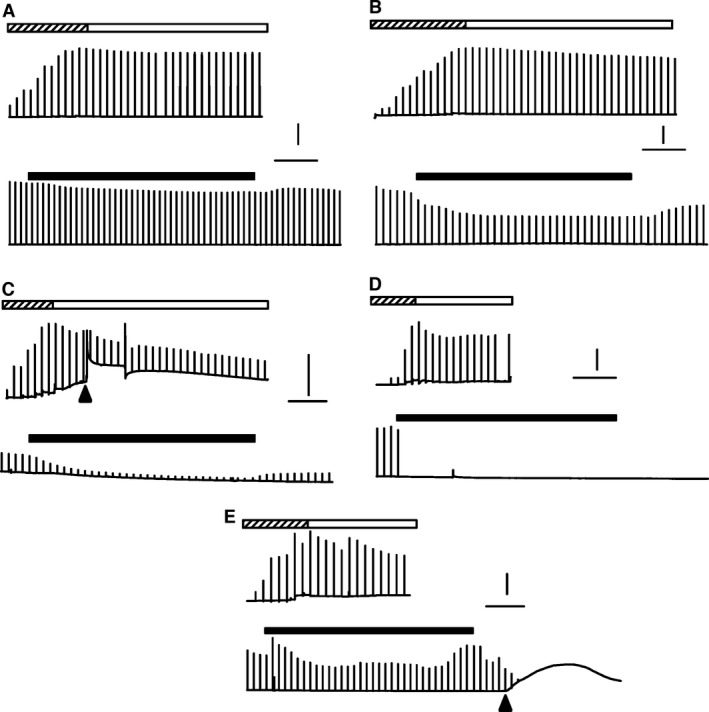
Examples of changes in unstimulated and tetanic force over time from (A) wild type EDL, (B–E) HyperKPP EDL. Chart recorder like force traces were generated by averaging three digitized force measurements every 2 sec and plotting a vertical line from minimum to maximum value when a contraction was elicited. Top traces were recorded while muscle length was adjusted to give maximum tetanic force (hatched horizontal bars) followed by a 30 min equilibrium period (white horizontal bars). Lower traces show the effect of raising [K^+^]e from 4.7 to 11 mmol/L (black horizontal bars) followed by recovery at 4.7 mmol/L K^+^. Muscles were elicited to contract every 100 sec. Arrow heads indicate when a sudden contracture occurred; horizontal lines represent 10 min; vertical lines represent 5 g of force. HyperKPP, hyperkalemic periodic paralysis.

The weakness and paralytic attacks in HyperKPP arise from a greater sensitivity to K^+^‐induced force depression (Hayward et al. 2008; Clausen et al. 2011; Lucas et al. 2014; Ammar et al. 2015). Increasing [Ca^2+^]e reduces the extent of the K^+^‐induced force depression in wild type and HyperKPP muscles (Lucas et al. 2014; Cairns et al. 2015). The aim here was to assess whether the extent of the K^+^‐induced force depression and Ca^2+^‐alleviation change as HyperKPP mice age. Experiments were carried out using the symptomatic fast‐twitch glycolytic EDL and slow‐twitch oxidative soleus. Every experiment started with the adjustment of muscle length followed by a 30 min equilibrium period while [K^+^]e was 4.7 mmol/L. For wild‐type EDL, muscle lengthening gave rise to smooth increases in peak force until maximum tetanic force was reached with very small increases in baseline; that is, unstimulated force (Fig. 8A). Tetanic force was relatively stable during the equilibrium period and decreased little when [K^+^]e was increased from 4.7 to 11 mmol/L.

Paralytic attacks in human patients are unpredictable with large variation in frequency and severity among patients (Gamstorp et al. 1957; Charles et al. 2013). This phenotype was obvious with the force measurements as shown with four examples (Fig. 8B–E). Some HyperKPP EDL behaved like wild type with the exception of a greater decrease in peak tetanic force at 11 mmol/L (Fig. 8B). Some HyperKPP EDL had large increases in unstimulated force while being stretch (Fig. 8C), and in that example a sudden contracture occurred in the absence of any stimulation or stretching followed by a significant loss in tetanic force. Some HyperKPP EDL completely stopped contracting upon stimulation when [K^+^]e was increased to 11 mmol/L K^+^ with no recovery when [K^+^]e was returned to 4.7 mmol/L (Fig. 8D). In a last example, a contracture occurred when [K^+^]e was returned to 4.7 from 11 mmol/L with a complete loss to contract when stimulated (Fig. 8E).

#### Tetanic force

**Figure 9:**
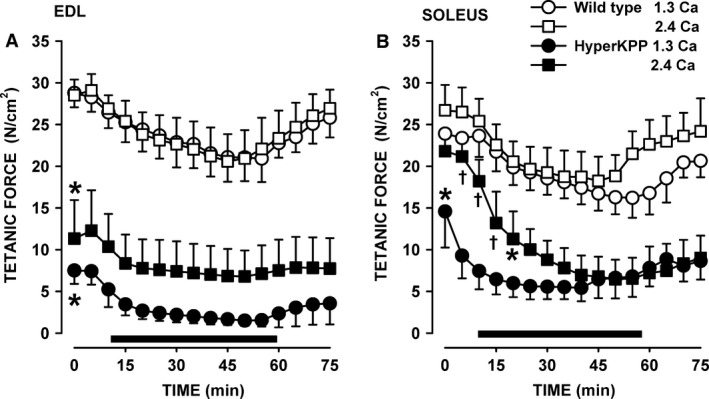
HyperKPP (A) EDL and (B) soleus had significantly lower mean tetanic force than wild‐type muscles, especially at low [Ca^2+^]e. EDL and soleus were from 4‐month‐old mice. Horizontal black bar indicates the time at (A) 11 mmol/L K^+^ and (B) 10 mmol/L. Vertical bars are the SE of five muscles. *Indicates when mean tetanic force of HyperKPP muscles became significantly less than that of wild type. ^†^Mean peak tetanic force at 2.4 mmol/L Ca^2+^ was significantly different from the force at 1.3 mmol/L Ca^2+^; ANOVA and LSD, *P* < 0.05. LSD, least square difference; ANOVA, analyses of variance; HyperKPP, hyperkalemic periodic paralysis.

Figure 9 shows how mean tetanic force changed over time at elevated [K^+^]e for muscles from 4‐month‐old mice. For EDL, mean tetanic forces were significantly lower in HyperKPP than in wild type at both 4.7 and 11 mmol/L K^+^ (Fig. 9A). While Ca^2+^ had no effect on mean tetanic force of wild‐type EDL, it improved mean tetanic forces in HyperKPP EDL although the differences between 1.3 and 2.4 mmol/L Ca^2+^ were not significant due to a large variation at 2.4 mmol/L Ca^2+^. Wild‐type soleus muscle is more sensitive to an increase in [K^+^]e than EDL (Cairns et al. 1997; Lucas et al. 2014). So, for this muscle the K^+^ challenge was set to 10 mmol/L. At 1.3 mmol/L Ca^2+^, HyperKPP soleus generated significantly lower mean tetanic force than wild type at both 4.7 and 10 mmol/L K^+^ (Fig. 9B). At 2.4 mmol/L Ca^2+^, on other hand, mean tetanic force was lower in HyperKPP than in wild‐type soleus but the difference was not significant. Increasing [K^+^]e to 10 mmol/L caused larger decreases in mean tetanic force in HyperKPP than wild‐type soleus; so, over time tetanic force became significantly less in HyperKPP. A return of [K^+^]e from 10 or 11 mmol/L to 4.7 mmol/L allowed for a recovery of mean tetanic force in wild type EDL and soleus, whereas in HyperKPP muscles the extents of force recovery were much smaller.

**Figure 10:**
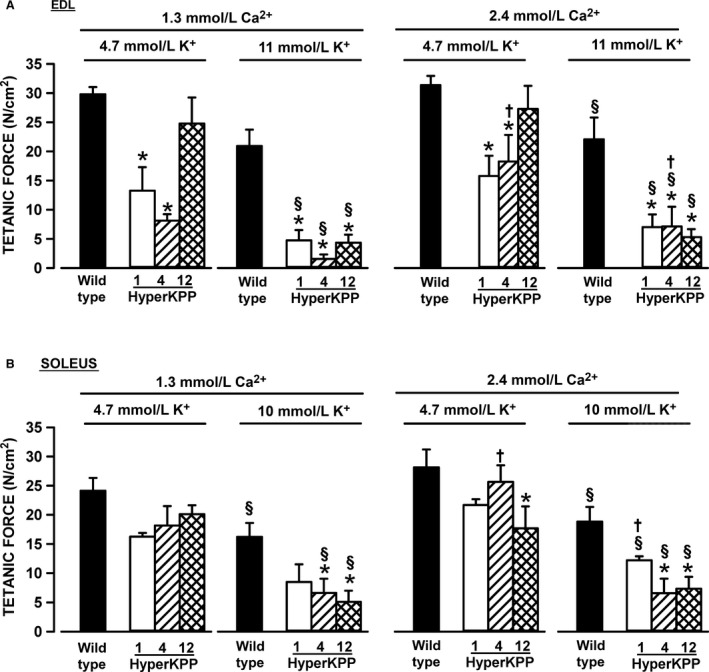
The sensitivity of HyperKPP (A) EDL and (B) soleus did not change between the ages of 1–12 months. Vertical bars represent the SE of five muscles. *Mean tetanic force of HyperKPP muscle significantly different from that of wild type. ^§^Mean tetanic force at 11 mmol/L K^+^ significantly different from that at 4.7 mmol/L K^+^. ^†^Mean tetanic force at 1.3 mmol/L Ca^2+^ significantly different from that at 2.4 mmol/L Ca^2+^. ANOVA and LSD, *P* < 0.05. LSD, least square difference; ANOVA, analyses of variance; HyperKPP, hyperkalemic periodic paralysis.

Similar experiments were carried out on EDL and soleus from 1‐ and 12‐month‐old mice. For wild type, mean peak tetanic forces were not significantly different for EDL and soleus between the ages of 1, 4, or 12 months (data not shown), which is in agreement with a previous study (Gong et al. 2000). Overall, there was no evidence for a worsening of muscle weakness in HyperKPP EDL between 1 and 12 months (Fig. 10A). Interestingly at 4.7 mmol/L K^+^ and both [Ca^2+^]e levels, mean tetanic force of HyperKPP EDL from 12‐month‐old mice were higher compared to the force at 1 and 4 month, but were also not significantly different from the wild‐type mean tetanic force. However, increases in [K^+^]e to 11 mmol/L reduced mean tetanic force of HyperKPP EDL to similar levels with no significant difference between 1, 4, and 12 months. For soleus, there was no difference between the three ages regardless of the [K^+^]e or [Ca^2+^]e (Fig. 10B).

#### Unstimulated force

**Figure 11:**
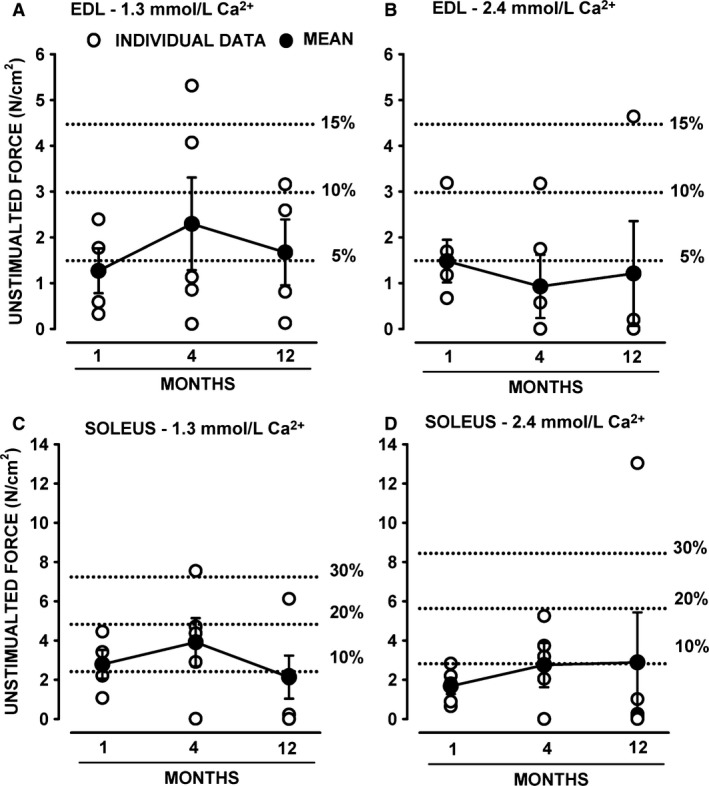
Many HyperKPP muscles developed large amount of unstimulated force. As shown in Figure 8, HyperKPP muscles often generated unstimulated force in the absence of stimulation when stretched or when they elicited a contracture. Large increases in unstimulated force could occur at any time during an experiment. Here, we report the mean and individual values for the largest unstimulated force measured during the course of an entire experiment. Dashed lines represent the amount of unstimulated force relative to the mean peak tetanic force of wild‐type EDL (A, B) and soleus (C, D). Vertical bars represent the SE of four to five muscles. HyperKPP, hyperkalemic periodic paralysis.

As shown in Figure 8, unstimulated force measured from the baseline was unstable in many HyperKPP muscles. To best illustrate the extent and variability of unstimulated force, we plotted both the mean and individual values of the highest unstimulated forces measured in the course of an experiment for each of the three ages. For HyperKPP EDL at 1.3 and 2.4 mmol/L Ca^2+^, mean unstimulated forces ranged from 0.9 to 2.3 N/cm^2^ for 1‐, 4‐, and 12‐month‐old mice whereas individual unstimulated forces ranged from 0.11 to 5.31 N/cm^2^ (Fig. 11A, B). Notably, the unstimulated force of 6 HyperKPP EDL was equal to or greater than 10% of the mean peak tetanic force of wild‐type EDL. In general, HyperKPP soleus generated more unstimulated force as mean unstimulated force ranged from 1.6 and 4.5 N/cm^2^ and individual values between 0.1 and 13.0 N/cm^2^ (Fig. 11C, D). A total of 7 HyperKPP solei had unstimulated force that was close to or greater than 20% of the mean tetanic force of wild‐type soleus.

### Fiber type

**Figure 12:**
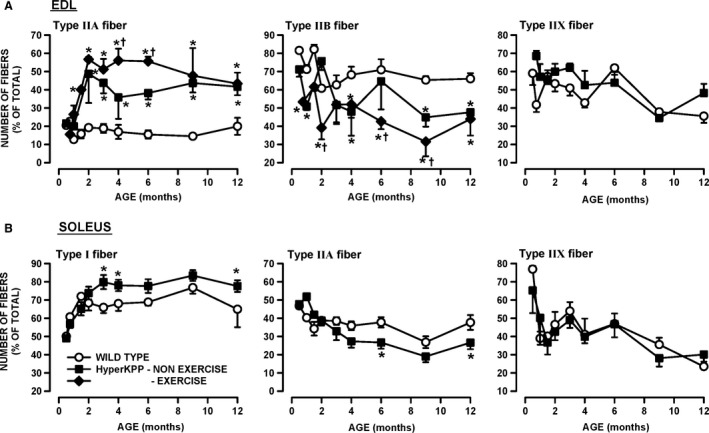
Significant change in myosin isoform expression occurred in HyperKPP (A) EDL and (B) soleus when compared with wild‐type muscles. The number of fibers expressing each of the myosin isoform was determined in EDL and soleus of nonexercise and exercise wild type and HyperKPP mice. The exercise mice were the same as those used for the treadmill running in Figure 3. Treadmill running had no significant effect on the number of fiber expressing each of the four myosin isoforms in wild‐type EDL/soleus and HyperKPP soleus as well as the number of fibers expressing myosin IIX in HyperKPP EDL (ANOVA and LSD, *P* > 0.05). For clarity, the data from nonexercise and exercise mice were pooled for those conditions and the differences between exercise and nonexercise mice in the number of fibers expressing myosin IIA and IIB are only shown for EDL HyperKPP mice. The numbers of fibers expressing myosin I in EDL and myosin IIB in soleus are not shown as they were less than 1%. Vertical bars represent the SE of six to eight mice. *Mean number of fibers for HyperKPP muscles significantly different from that of wild type. ^†^Mean number of fibers for exercise mice significantly different from that of nonexercise mice, ANOVA and LSD *P* < 0.05. LSD, least square difference; ANOVA, analyses of variance; HyperKPP, hyperkalemic periodic paralysis.

A shift in fiber type from glycolytic myosin IIB to more oxidative myosin IIA fibers is another HyperKPP phenotype (Hayward et al. 2008). Here, we determined when the shift starts and completes. For these measurements, we collected EDL and soleus muscles from nonexercise mice as well as from the same mice that were elicited to run on treadmill (Fig. 3). EDL fibers from 3‐week to 12‐month‐old wild‐type mice as well as HyperKPP EDL from 3‐ to 4‐week‐old mice expressed primarily myosin IIA (~20%), IIB (60–70%) and IIX (~60%) isoforms (Fig. 12A–C); very few fibers (<2%) expressed myosin I isoform (data not shown). It is important to note that the sum of all percent value exceeded 100% because we counted the number of fibers expressing each of the myosin isoforms and many fibers expressed more than one myosin isoforms as previously reported (Banas et al. 2011). A significant increase in the number of fibers expressing myosin IIA occurred between the 1st and 2nd month, whereas significant decreases in the number of fibers expressing myosin IIB ended by the 3rd month. Remarkably, in some cases the 5‐day treadmill running caused significant further increases in the number of fibers expressing myosin IIA and decreases in the number of fibers expressing myosin IIB in HyperKPP EDL; an effect not observed for wild‐type EDL. Finally, the number of fibers expressing more than one myosin isoforms increased in HyperKPP EDL as the sum of the number of fibers expressing each of myosin IIA, IIB, and IIX was 158% compared to only 133% for wild‐type EDL from 2‐month‐old mice. A shift toward significant increases in the number of fibers expressing myosin I and significant decreases in the number of fiber expressing myosin IIA was also observed in HyperKPP soleus, but in this muscle treadmill running did not cause any further shift (data not shown).

**Figure 13:**
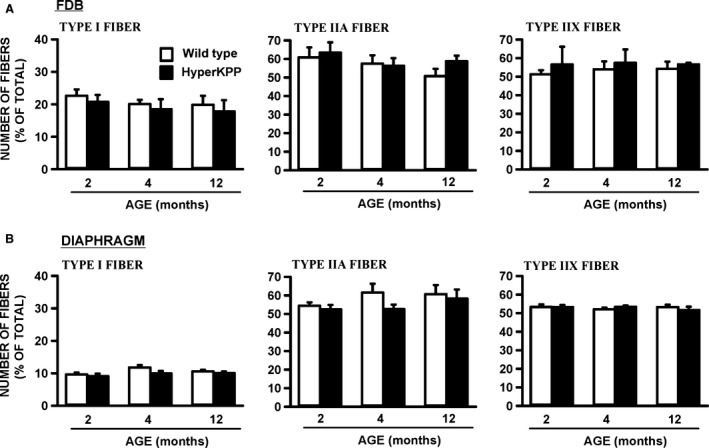
There was no difference in myosin isoform expression between wild type and HyperKPP (A) FDB and (B) diaphragm (ANOVA, *P* > 0.05). The number of fibers expressing myosin IIB was less than 5% in diaphragm and less than 1% in FDB (data not shown). Vertical bars represent the SE of four to eight mice. ANOVA, analyses of variance; HyperKPP, hyperkalemic periodic paralysis.

The shift in fiber type composition in EDL and soleus is most likely related to the greater number of action potentials being generated by muscle fibers in the absence of motorneuron stimulation that maintain high [Ca^2+^]i, which in turn modulates the expression of various myosin isoforms (Chin et al. 1998; Dunn et al. 1999). In a previous study (Lucas et al. 2014), we provided evidence that the diaphragm and FDB of HyperKPP mice are asymptomatic. Here, we report that the fiber type composition of both HyperKPP muscles remained unchanged up to 12 months of age further supporting the notion that neither muscles suffer of HyperKPP symptoms, at least in terms of myotonic discharge (Fig. 13).

### Fiber damage

**Figure 14:**
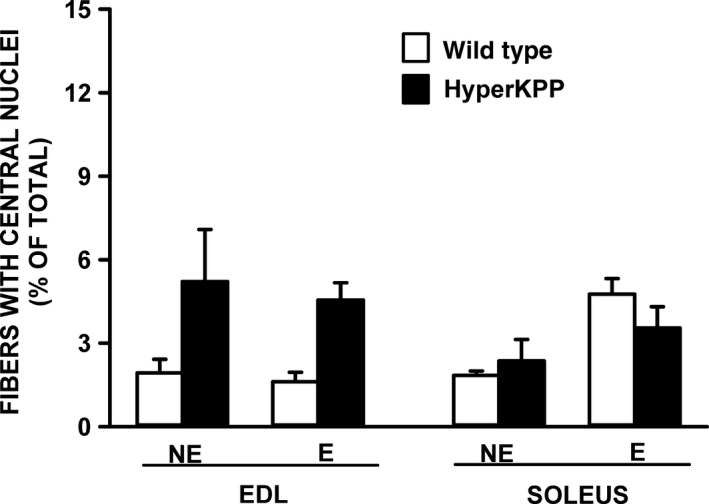
The number of EDL fibers containing central nuclei was not significantly different between 12‐month‐old wild type and HyperKPP. Mice were either from the nonexercise (NE) or exercise (E) group. The exercised mice were the same as those used for the treadmill running in Figure 3. The presence of central nuclei was determined from 10 *μ*m cross sections stained with H&E. The number of fibers containing central nuclei were counted and expressed as a percent of the total number of fibers. No significant difference, ANOVA and LSD, *P* < 0.05. LSD, least square difference; ANOVA, analyses of variance; HyperKPP, hyperkalemic periodic paralysis.

Individuals with HyperKPP often develop fixed muscle weakness in middle age associated with internalized muscle nuclei and irreversible vacuolar myopathy (Pearson 1964; Bradley et al. 1990; Miller et al. 2004). For wild‐type mouse EDL, internal nuclei became apparent by 12 months of age but only in 2% of all fibers in exercised and nonexercised mice (Fig. 14). The fraction of fibers with internal nuclei appeared slightly higher in HyperKPP EDL, but the difference compared to wild‐type muscle was not significant. No difference in morphology was observed between wild type and HyperKPP soleus. Considering the low level of fibers of internal nuclei in muscles from 12‐month‐old mice, no analysis was carried out with muscles from younger mice.

## Discussion

This study provides novel evidence for (i) increased EMG activity and attacks of immobility in the M1592V HyperKPP mouse model during freely moving conditions; (ii) decreased running capacity in young (<3‐month old) HyperKPP mice; (iii) a time course for changes in Na_V_1.4 channel content, TTX‐sensitive Na^+^ influx, and changes in contractile characteristics showing that HyperKPP symptoms maximally impact on muscle contractility at a time when NaV1.4 channel content and Na^+^ influx reach their mature levels.

### The cause of muscle stiffness and weakness in M1592V HyperKPP

As described in the Introduction, a key phenotype of 21 HyperKPP patients from an English family with the M1592V mutation included mild monthly attacks consisting of muscle stiffness with no myotonia, except upon penetration or movement of the concentric EMG needle. Our EMG measurements provided evidence for greater electrical activity in the gastrocnemius of M1592V mouse than in wild type. First, the total iEMG was greater in HyperKPP gastrocnemius especially when mice were active. Second, the duration versus diEMG/dt relationship was shifted toward higher diEMG/dt values and the duration for which diEMG/dt was above 1200 *μ*V·sec/min was 2.4 times longer in HyperKPP. Third, peak EMG amplitudes during an activity burst were greater in HyperKPP. The greater total iEMG, diEMG/dt, and burst amplitude suggest that HyperKPP gastrocnemius fibers generate more action potentials than wild type. The question is whether HyperKPP fibers received more neuronal stimulation compared to wild type or because they themselves generated action potentials in the absence of neuronal stimulation?

Sudden contractions occurred during muscle dissection and between electrically elicited tetanic contractions during an experiment (Fig. 8); that is, they occurred in the complete absence of electrical or neuronal stimulations. Considering that HyperKPP involves a defect in membrane excitability due to defective Na_V_1.4 channels, it is unlikely that these contractions are electrically silent; that is, related to sudden Ca^2+^ release in the absence of any generation of action potentials. We therefore suggest that the sudden contractions in vitro and the greater EMG activity in HyperKPP gastrocnemius are due to periods of cell membrane hyperexcitability during which action potentials are generated by muscle fibers themselves in the absence of any stimuli.

Clinically, myotonia is defined as a sustained burst of action potentials, originating from the muscle fiber itself, persisting for several seconds after motor neuron activity has ceased (Cannon 2006). Myotonic discharges can be elicited in skeletal muscle of mouse and patient with the M1592V mutation upon electrodes penetrations or movements (Hayward et al. 2008; Chinnery et al. 2002). However, when free to move, a sustained series of short EMG peaks as expected for a myotonic run was observed for one brief period and from only one HyperKPP mouse (Fig. 7E). Furthermore, while the amplitudes of EMG activity bursts were higher in HyperKPP, their mean durations were similar to those of wild type. Finally, relaxation times following contractions were of the same duration in wild type and HyperKPP muscles (this study, data not shown; Hayward et al. 2008). These results suggest that myotonic discharge is not a key feature in the M1592V mouse model as reported for one family with the same mutation (Poskanzer and Kerr 1961; Chinnery et al. 2002). We therefore suggest that the M1592V mutation results in periods of hyperexcitability during which HyperKPP fibers randomly generate action potentials and the resulting contractions are the cause of the muscle stiffness reported by patients.

A second key phenotype of 21 HyperKPP patients from an English family with the M1592V mutation included muscle weakness lasting 3–4 days, but rarely a full paralytic attack (once or twice a year). Muscle weakness was also evident in HyperKPP soleus and EDL as they generate less tetanic force than their wild‐type counterparts even at normal [K^+^]e (this study; Hayward et al. 2008; Lucas et al. 2014). We recently reported that at 4.7 mmol/L K^+^ the frequency distribution of resting membrane potential of HyperKPP soleus and EDL fibers was shifted toward less negative potential when compared with wild‐type fibers, with a large proportion of fibers with resting EM less negative than −55 mV, a potential at which muscle fibers no longer generate action potentials (Ammar et al. 2015). The unstimulated force observed during in vitro force measurements rarely exceeded 15% of maximal force in EDL (or 30% in soleus) suggesting that few fibers at a time become hyperexcitable. The resulting action potentials are then expected to trigger local increases in interstitial [K^+^] and not necessarily across the entire muscle reducing force generation only in fibers exposed to higher [K^+^] as HyperKPP fibers have a greater sensitivity to K^+^‐induced force depression; that is, the localized increase in [K^+^]e does not cause full muscle paralysis but instead just a weakness even when muscles are exposed to 4.7 mmol/L K^+^. This may also help to explain why the M1592V mutant causes symptoms under normokalemic plasma conditions (Poskanzer and Kerr 1961).

Hindlimb immobility was observed with the M1592V mouse when mice were transferred to new cages and at random before, during and after treadmill running. Prolonged hindlimb immobility as well as prolonged periods of reduced EMG activity in M1592V HyperKPP gastrocnemius were not a major phenotype in the M1592V mouse, being consistent with the fact that severe paralytic attacks occur only infrequently in M1592V patients. We could not observed hindlimb immobility during any of the 4 h of EMG recording in many HyperKPP mice, so it is not possible to clearly establish its cause. The I588V HyperKPP mouse model also suffers of hindlimb immobility “lasting a few seconds”; remarkably, the immobility was associated with an increased EMG activity (Corrochano et al. 2014). Periods of reduced EMG activity were not reported for the I588V model, but did occur in the M1592V mouse. Moreover, hindlimb immobility, burst amplitude and reduced EMG activity were less frequent at 6 than at 1–2 months of age. Hindlimb immobility in the M1592V model is thus more likely associated with EMG activity bursts followed by reduced EMG activity (or paralysis). Another feature of the hindlimb mobility was its frequency, being the highest during the first month of age and then being a rare occurrence thereafter. The reason for this cannot be explained from our results. However, HyperKPP patients can sense an upcoming weakness or paralytic attack and sometimes prevent it with mild exercise (Poskanzer and Kerr 1961). Perhaps, HyperKPP mice are also learning how to avoid hindlimb immobility after one or 2 months of age.

A final feature of the human M1592V phenotype is the lack of any respiratory distress (Poskanzer and Kerr 1961) even though (i) human and mouse diaphragm expresses the NaV1.4 channel and (ii) HyperKPP diaphragm has similar TTX‐sensitive Na^+^ influx compared to EDL and soleus (Zhou and Hoffman 1994; Lucas et al. 2014). One reason for the lack of distress is because HyperKPP diaphragm does not suffer of greater K^+^‐induced force depression like EDL and soleus do when compared with their wild‐type counterpart (Lucas et al. 2014; Ammar et al. 2015). Another reason may be that diaphragm muscle fibers do not generate any action potential in the absence of stimulation as hindlimb muscles do. Contrary to HyperKPP EDL and soleus, there was no alteration in fiber type composition in HyperKPP diaphragm up to the age of 12 months. As suggested by Hayward et al. (2008), the fiber shift in EDL and soleus is most likely triggered by increased muscle activity associated with the greater amounts of action potentials generated in the absence of any stimulation mimicking the effects of resistance training. Thus, the lack of any fiber type transition in diaphragm suggests that it does not experience membrane hyperexcitability as hindlimb muscles do.

### Running capacity of HyperKPP mice

Up to 3 months of age, HyperKPP mice have lower running capacity compared to wild type as they failed sooner in maintaining the required work load. As discussed above, active HyperKPP mice have greater iEMG as they generate more action potentials, which then results in three major consequences. First, more Ca^2+^ is released by the sarcoplasmic reticulum imposing a greater metabolic demand by Ca^2+^ ATPase pump. Second, when extra action potentials are generated, the resulting contractures as observed in vitro then require greater force generation between antagonistic muscles, which again increases metabolic demand. The increase in metabolic demand then most likely results in the development of metabolic stress triggering fatigue sooner in HyperKPP than in wild‐type muscles. Third, more action potentials may give rise to greater increases in interstitial [K^+^] than normal. HyperKPP muscles being more sensitive to the K^+^‐induced force depression, they then become weak lowering the running capacity.

By the age of 4 month old, HyperKPP mice started to have greater running capacity than wild type. It is very likely that the greater muscle oxidative capacity in HyperKPP than wild‐type mice (Hayward et al. 2008) plays a role in reducing the impact of the extra metabolic demand discussed above. However, the differences in running capacity between wild type and HyperKPP were small. Two‐month‐old wild‐type mice increases their running capacity at least threefold over a 35‐day period of running 5 days a week (Thabet et al. 2005). So, even though older HyperKPP mice had greater running capacity than wild type because of an increase oxidative capacity, the latter factor was still not sufficient to significantly improved running capacity.

### Implication of our finding for the progression of HyperKPP symptoms in patients

Patients suffering of HyperKPP due to the M1592V mutation have a first paralytic attack usually between the age of 5 and 10 years, and the frequency and severity of attacks increase until adolescence (Poskanzer and Kerr 1961; Miller et al. 2004). Considering the difference in human and mouse lifespan, three questions were raised in the Introduction: (i) how do symptoms progress in the M1592V HyperKPP mouse model?, (ii) at what ages should we study the disease in the mouse model?, and (iii) is the severity of HyperKPP symptoms in terms of membrane excitability defects linked to the NaV1.4 mutant expression or does it continue to worsen after the channel content has reached adult level?

The total NaV1.4 channel content in adult HyperKPP EDL and soleus are, respectively, 54% and 73% of the content in wild type (Lucas et al. 2014). Here, we report that the postnatal increase in NaV1.4 channel content reached the adult level in 3 weeks in wild type and HyperKPP hindlimb muscles, whereas the abnormally high TTX‐sensitive Na^+^ influx in HyperKPP EDL and soleus reached a maximum at 2 weeks of age. Measurements of hindlimb immobility, EMG activity, running capacity, unstimulated and tetanic force did not provide evidence for any major worsening of membrane excitability and contractile defects in HyperKPP EDL and soleus past the age of 3 weeks up to 12 months. The only exception was the fiber type transition, which for EDL and soleus was completed at 2 and 3 months of age, respectively. However, as discussed above, the changes in fiber‐type composition is most likely associated with extra muscle activity due to more action potential being generated. It then implies that the change in fiber type can only occur sometimes after the onset of the defect in membrane excitability. So, our results do not provide evidence for a worsening of HyperKPP symptoms once the Na_V_1.4 channel content has reached adult level.

A lack of disease progression in M1592V HyperKPP mouse represents the first phenotypic difference with human patients. However, it does not necessarily imply a species difference. In mouse and rat, the embryonic Nav1.5 transcript is no longer expressed by 3–4 weeks of age whereas the NaV1.4 mRNA transcript and protein contents have reached adulthood level (this study; Lupa et al. 1993; Sherman and Catterall 1982; Zhou and Hoffman 1994). In human, the NaV1.4 mRNA content at age 5 is only 30% of the adult level (Zhou and Hoffman 1994) suggesting that NaV1.4 channel protein content may also be much lower than in adult. More importantly, the largest increase in Na_V_1.4 channel content and the onset and worsening of HyperKPP symptoms occur after the age of 5 years. If the situation is the same in patients and mice, then it would appears that (i) the NaV1.4 channel content up to 5 years of age may be in most cases too low to trigger symptoms in patients with the M1592V mutation and (ii) symptom worsening during adolescence can then be largely due to an increase in the Na_V_1.4 channel content toward adult levels. It must be kept in mind, however, that other changes in protein expression concomitantly to the increase in Na_V_1.4 channel may also contribute to the symptoms worsening. So, future studies are still necessary to fully understand whether symptom emergence depends on a threshold Na_V_1.4 content and whether symptom worsening during adolescence is solely a function of the increase in Na_V_1.4 content.

In conclusion, this study demonstrated that the M1592V HyperKPP mouse between the ages of 1–12 months represents a good model to study the HyperKPP mechanisms in patients carrying the M1592V mutation as adult mouse and human patients shared several common physiological phenotypes; the only exception is the lack of symptom progression in the mouse model possibly because Na_V_1.4 channel protein content reaches adult levels in 3 weeks in mouse and in more than 5 years in human. The HyperKPP phenotype for the M1592V mouse model reached its final stage at the time when the total NaV1.4 channel content reached its adult level. Our results did not provide evidence that the phenotypic behavior of M1592V HyperKPP hindlimb muscles worsened once the Na_V_1.4 channel has reached adult level suggesting that the symptoms are a consequence of the defective NaV1.4 channels and possibly any changes in protein expression during the first postnatal 3 weeks. Accordingly, it is suggested that the delayed onset and worsening of HyperKPP symptoms until adolescence in human patients may be a function of the NaV1.4 channel content that requires more than 5 years to reach adult levels.

## Conflict of Interest

The authors declare no competing financial interests.
